# Precise Detection, Control and Synthesis of Chiral Compounds at Single-Molecule Resolution

**DOI:** 10.1007/s40820-023-01184-5

**Published:** 2023-09-12

**Authors:** Chen Yang, Weilin Hu, Xuefeng Guo

**Affiliations:** 1 Beijing National Laboratory for Molecular Sciences, National Biomedical Imaging Center, College of Chemistry and Molecular Engineering, Peking University, 292 Chengfu Road, Haidian District, Beijing, 100871 People’s Republic of China; 2 Center of Single-Molecule Sciences, Institute of Modern Optics, Frontiers Science Center for New Organic Matter, College of Electronic Information and Optical Engineering, Nankai University, 38 Tongyan Road, Jinnan District, Tianjin, 300350 People’s Republic of China

**Keywords:** Single-molecule junction, Molecular chirality, Photonic polarization, Electronic spin, Asymmetric reaction

## Abstract

Chirality, as the symmetric breaking of molecules, plays an essential role in physical, chemical and especially biological processes, which highlights the accurate distinction among heterochiralities as well as the precise preparation for homochirality. To this end, the well-designed structure-specific recognizer and catalysis reactor are necessitated, respectively. However, each kind of target molecules requires a custom-made chiral partner and the dynamic disorder of spatial-orientation distribution of molecules at the ensemble level leads to an inefficient protocol. In this perspective article, we developed a universal strategy capable of realizing the chirality detection and control by the external symmetry breaking based on the alignment of the molecular frame to external stimuli. Specifically, in combination with the discussion about the relationship among the chirality (molecule), spin (electron) and polarization (photon), i.e., the three natural symmetry breaking, single-molecule junctions were proposed to achieve a single-molecule/event-resolved detection and synthesis. The fixation of the molecular orientation and the CMOS-compatibility provide an efficient interface to achieve the external input of symmetry breaking. This perspective is believed to offer more efficient applications in accurate chirality detection and precise asymmetric synthesis via the close collaboration of chemists, physicists, materials scientists, and engineers.

## Highlights

- Single-molecule electrical detection, especially the single-molecule junction setup, enables the precise detection and spatial operability of anchored molecules.
- The transition among asymmetric characteristics (i.e., molecular chirality, photonic polarization and electronic spin) is proposed as a universal methodology to realize the detection, control and synthesis of chirality.
- Exploring the origin of symmetry breaking contributes to the development of a general reliable strategy for asymmetric synthesis.

## Graphical abstract

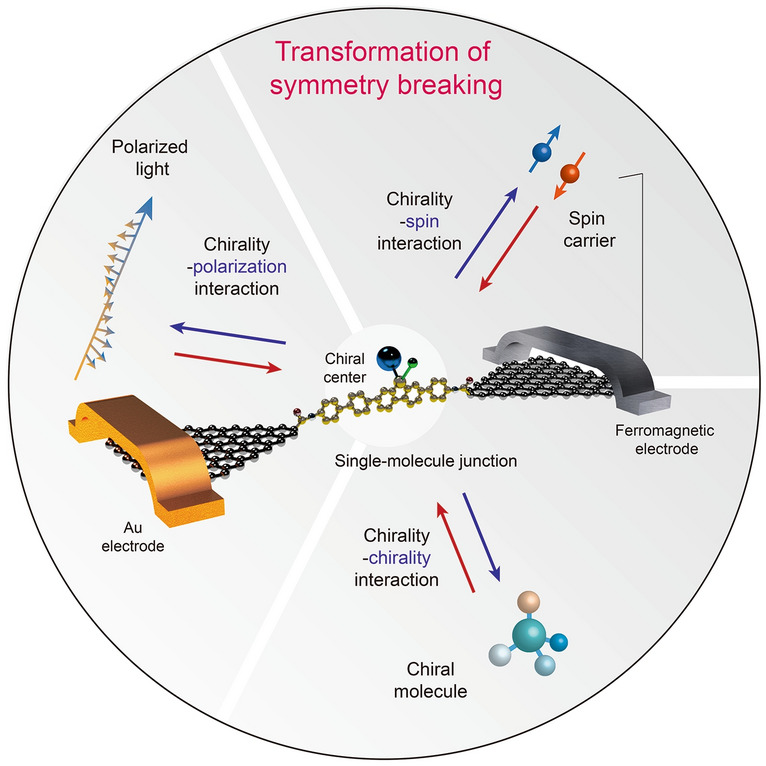

## Introduction

**Fig. 1:**
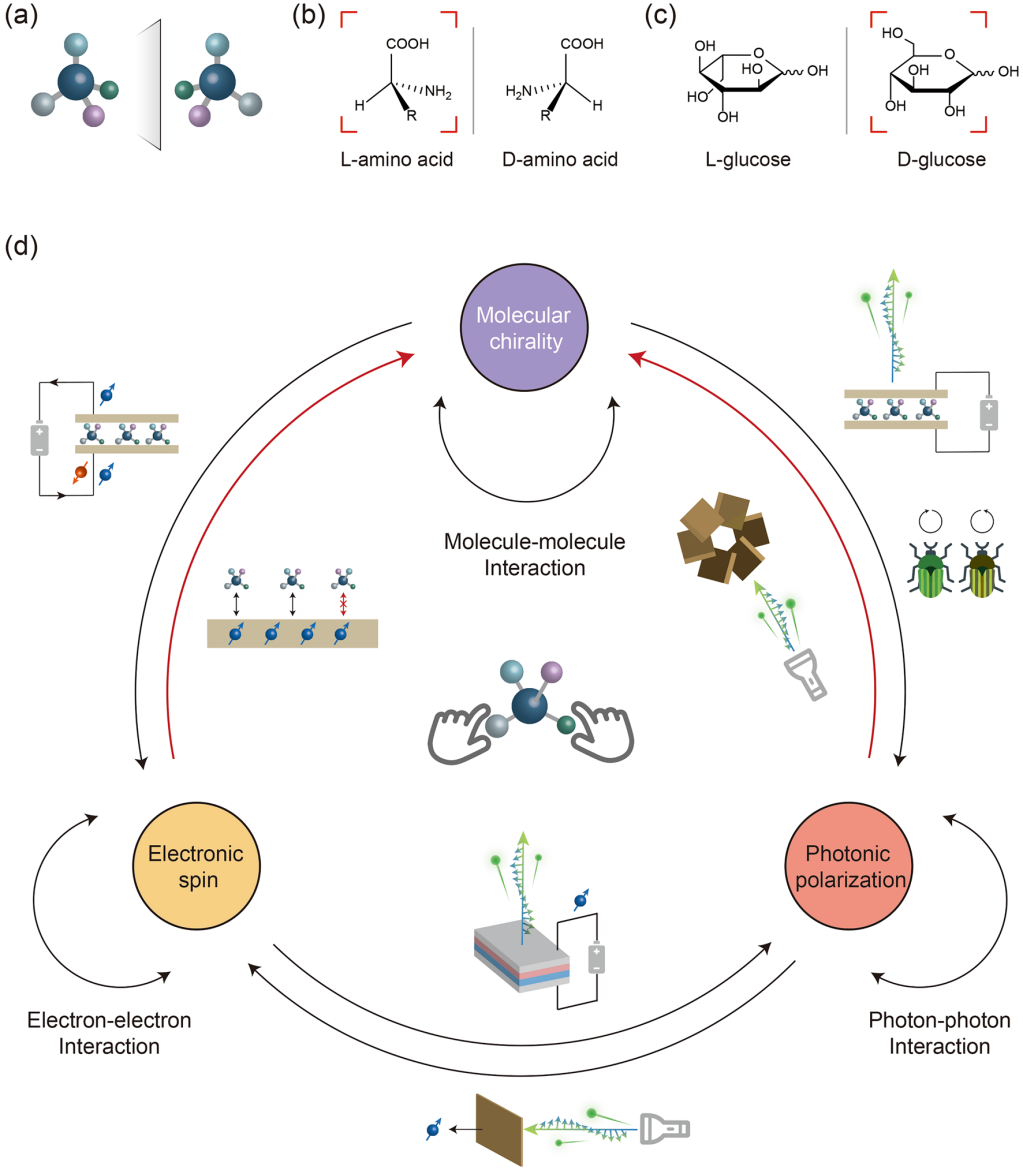
Schematic illustration of molecular chirality, electronic spin and photonic polarization. **a** Schematic of the chiral molecules (enantiomers) with a mirror relationship. **b** The chiral amino acids, where the natural l-configuration was highlighted. **c** The chiral glucoses, where the natural d-configuration was highlighted. **d** The relationship among the molecular chirality, electronic spin and photonic polarization

Chirality is a descriptor of the molecular spatial structure. Two chiral individuals, although with identical composition, they are not superimposable and show a mirrored relationship, just like the right and left hands (Fig. 1a). These two conformations, i.e., the enantiomers, are intuitively equivalent in the physical, chemical, and biological processes. However, a chirality preference can be traced back to the early earth [1, 2], e.g., the l-amino acid (Fig. 1b) and d-carbohydrate (Fig. 1c), which results in a strict selectivity to the chirality during the life process and a rigorous consideration of chirality in the pharmaceutical industry nowadays. Furthermore, the chirality was also found as a key factor to determine the performance of functional materials [3, 4]. Therefore, the detection and control of the chirality is an ongoing major project of research communities. Considering the inherent complexity of the chemical structure, it is a non-trivial task.

The other symmetry breaking elements in the nature, including the polarized light and spin electron, are inextricably associated with the chirality and provide new insights into addressing the above challenges (Fig. 1d). For example, the polarized light can be reflected by the exoskeleton of beetle via an array of chiral mesogens [5]. The spin-filtering effect was also observed when the polarized electron transports through the chiral molecules [4, 6–8] (Fig. 1d). The interactions between electronic spin and photonic polarization are also very common. For example, the emission of polarized light could be realized by spin injection [9], and spin carriers could be generated in semiconductors by irradiation of polarized light [10]. Here, the interactions between spin current and polarized light are not discussed in detail. We mainly question whether it might be possible to utilize this concept to manipulate chirality by polarized light and spin electron. Therein, the key is to efficiently construct the interface to couple the different symmetry breaking characteristics. The determined orientation of the chiral molecules affords the capability to regulate the polarization of light and electron at a particular direction in the aforementioned cases. However, in general asymmetric synthesis, the dynamic disorder of the prochiral molecule in a homogeneous-phase solution could not totally meet the requirement to align the molecular frame to the introduced external polarized light and spin electron and therefore leads to an inefficient protocol.

To this end, the fixation of the molecule may be regarded as a promising strategy (Fig. 1d, the central inset). Trapping individual molecules provides a determined orientation and an opportunity to couple with external symmetry breaking. An additional benefit is the single-molecule and single-event precision for chirality detection and control, which obtain much more information beyond the ensemble average [11, 12]. In this aspect, we should appreciate the advances in single-molecule techniques in recent years, including confining molecules at surface [13–15], light trap [16], nano cavity [17, 18] or electrodes pair [19, 20], and detecting the corresponding single-molecule optical [13–15, 21], mechanical [16, 22, 23] and electrical signals [11, 18]. Among them, we believe that the fixation to at least two terminals of the molecules is particularly useful, i.e., the construction of the single-molecule junction by integrating them into electrodes. This setup enables the manipulation of the orientation of the trapped molecule. Meanwhile, the introduction of the optical and electrical inputs is also compatible.

Two typical single-molecule junctions classified by the movability of the electrode should be highlighted here, including the breaking junction [24, 25] and fixed junction [26–28]. Two typical examples of breaking junctions are scanning tunneling microscopy breaking junctions [24] and mechanically controllable breaking junctions [25]. Repeated contact and stretching of electrode pairs can build single-molecule junctions in nano-scale gaps repetitively. Considering the pertubed orientation of molecules between movable electrodes in breaking junctions, the fixed electrodes may be an alternative candidate, including the electro-migration junctions [26, 29] and the carbon-based single-molecule junctions [28, 30]. Their on-chip setup provides the CMOS-compatibility and enables the guidance of polarized light and the injection of spin electron conveniently. Therein, the graphene-molecule-graphene single-molecule junctions (GMG-SMJs) have a more stable covalent-bonded molecule-electrode interface. Therefore the orientation of the integrated molecule can be constrained strictly and manipulated by varying the chip’s orientation. In addition, the stable electrode and interface provide a high tolerance to complex reaction conditions, including the solvent, pH, temperature and voltage, thus paving a precise way to chirality detection and asymmetric synthesis.

In this perspective, we firstly summarized the typical techniques for chiral detection and control (enrichment). Then, by discussing the inextricably association among the natural symmetry breaking including the chiral molecule, spin electron and polarized light, we further pointed out the missing link among these characteristics: from optical polarization and electrical spin to molecular chirality. According to this, a series of feasible exploratory attempts for universal chirality detection and asymmetric synthesis via the single-molecule junction were proposed. Finally, these perspectives prompted the deliberations of the origin of the symmetry breaking, especially the chirality, which implies the existence of more unexplored strategies to achieve chiral detection and control fundamentally, urging further studies of this field.

## Molecular Chirality–Molecular Chirality Interaction

The macroscopic chirality detection and control are mainly based on the weak interaction between target molecule and chiral partner, i.e., the transformation of symmetry breaking from chirality to chirality. For the chirality detection, a series of chiral partners were adopted, such as cyclodextrin [31], polymer [32], metal–organic frameworks (MOF) [33] and macrocyclic compound [34] with an inherent or artificial custom-made chirality. The difference between the enantiomers interacting with the above partners could be detected by HPLC [35], NMR [36], electrochemistry [37] and thermodynamic measurements [38], thus achieving the chirality detection. The chiral control (enrichment) could also be further achieved via this method. Two strategies should be highlighted here. One is the separation to the existed enantiomers, including the earliest resolution of sodium ammonium tartrate crystals by tweezers (molecular self as the chiral partner), and the current techniques based on the different interaction between the chiral partners, e.g., HPLC [39] and kinetic resolutions [40]. The other strategy is the direct asymmetric synthesis, based on the specific interaction between prochiral molecule and chiral partner at the transition state or the pre-reactive state. A myriad of the chiral catalysts (partners) were reported and then a set of methodologies to asymmetric synthesis were established, which benefited from the unremitting efforts of organic chemists. However, the weak interaction could be easily interfered by the environment or surrounding disordered molecules. In other words, the chiral detection limitation and enantiomeric excess (ee) of the asymmetric synthesis were determined by an ensemble-average results based on the thermodynamics and kinetics of the interaction. A typical example is a poor stereo-selectivity at a high temperature due to the thermal disturbance.

**Fig. 2:**
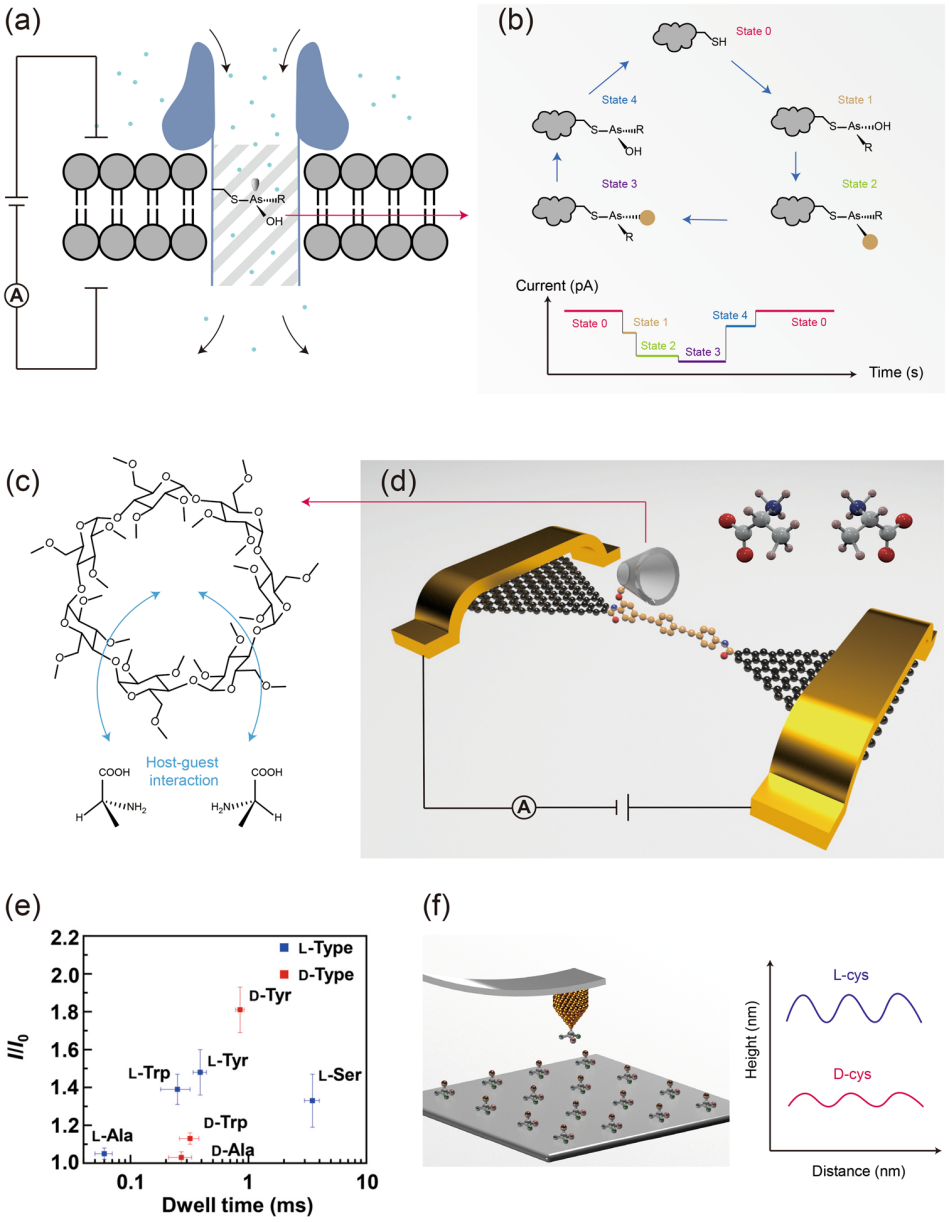
Chirality detection based on a chiral partner at the single-molecule level. **a** Schematic of the nanopore with a chiral site at the inner wall. **b** A typical conversion sequence of the monitored chiral species [41]. **c** Schematic of the host–guest interaction between the *β*-cyclodextrin and chiral amino acids. **d** Schematic of a graphene-based single-molecule junction with a *β*-cyclodextrin functional center to detect the chiral amino acids. **e** The fingerprints of the detected different amino acids with chirality [46]. Copyright 2021 The Authors, some rights reserved; exclusive licensee AAAS. Distributed under a CC BY-NC 4.0 license. **f** Schematic of the atomic force microscope with chiral-molecule-modified tips to detect the chiral molecules on the surface as well as the corresponding height-distance curves of the enantiomers. Panel **f** adapted from Ref. [47]. Copyright 2008 American Chemical Society

To address this challenge, a sensitive technique with single-molecule and single-event resolution is required for both the chirality detection and control. Based on the chirality-chirality symmetry breaking transformation, the target chiral molecules could be detected with different single-molecule approaches. A typical strategy is based on the host–guest interaction. For example, one small guest molecule in a nanopore could block the ionic current passing through (Fig. 2a) [41, 42]. The blocked current depends on the volume, shape and charge of the small molecules, which affords the single-molecule resolution. The chirality of the inner wall of the nanopore has a different interacting mode with individual enantiomers and then enables the resolvability of them by the ionic current. More importantly, the variation of the chirality during the S_N_2 nucleophilic substitution, i.e., the flip of the chirality before and after the reaction could be observed directly (Fig. 2b), which represents a breakthrough in sensitively detecting the chirality. Similarly, the single-molecule detection based on the single-molecule junction also affords the ability to detect the chirality. In this setup, the molecular conductance has a close relationship to its structure, configuration and conformation (Fig. 2d) [43–45]. To detect the chirality, our group adopted the *β*-cyclodextrin with natural chirality and integrated it into the graphene electrodes as a host molecule (Fig. 2c) [46]. The different amino acids, involving the chirality characteristics could be detected based on the interaction with *β*-cyclodextrin. The differences of the thermodynamic and kinetic during interaction could be detected by real-time measuring the conductance state and dwell time. This single-molecule approach conveniently mapped the characteristic fingerprints of different chiral amino acids, providing a promising route for high throughput detection (Fig. 2e). In addition, another single-molecule approach, such as the scanning probe microscope (SPM), could also detect the chirality with single-molecule insight [47]. At the case that the tips are silent to molecular chirality, they could be modified by a chiral partner and realize the chirality recognition of substrate molecules by measuring the chirality-related height or force (Fig. 2f). The single-molecule and single-event resolution reached the limit of the analytic chemistry and provided a more precise detection and even synthesis. However, this also requires the elaborate design of the corresponding chiral partner at each experiment, i.e., the artificial symmetry breaking.

## Molecular Chirality–Photonic Polarization Interaction

In fact, the natural symmetry breaking has also been applied to detect the chirality, such as the circular dichroism (CD) measured by polarized light, a universal and convenient method. Therein, the basic principle is the coupling of the symmetry breaking between photon and molecule. When polarized light passes through chiral matter, the propagation speed of left-polarized light is different from that of right-polarized light, which leads to the difference in refractive index and propagation speed of different polarized lights in chiral molecular media, which is also the basis of the relationship between molecular chirality and polarization. Arago et al. [48] found this optical rotation effect, which is often explained by the Fresnel hypothesis [49]: The linear polarized light propagating along the crystal optical axis can also be regarded as composed of two circular polarized lights with the same frequency and opposite rotation. In an optically active crystal, the two polarized light propagates at different speeds, resulting in rotation of the polarization plane through the chiral crystal. Such a hypothesis, although unable to explain the nature of the phenomenon, can convincingly explain the experimental results. Thereafter, the measurement of optical rotation became the main method for the identification of the two enantiomers. The detailed physical picture of the optical rotation necessitates the characterization at single-chiral-molecule scale in future.

**Fig. 3:**
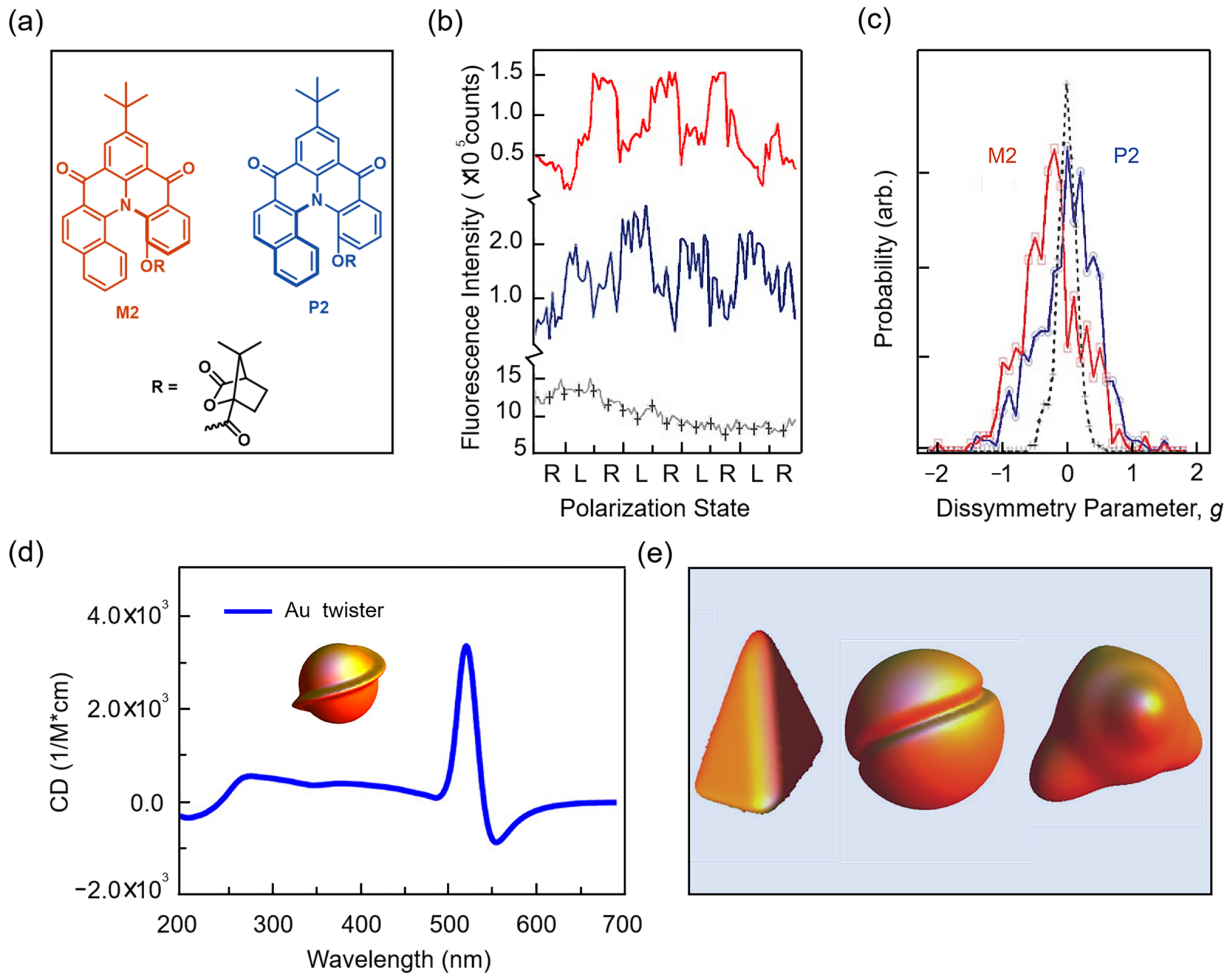
Direct chirality detection by CD. **a** Structures of M2 or P2 molecules [50]. **b** Representative fluorescence intensity traces versus the excitation polarization state (L or R). The black line is the dye-doped polymer nanosphere; The blue line is the P2 molecule; The red line is the M2 molecule. **c** Normalized histograms of fluorescence detection CD dissymmetry parameters, *g*, which can be determined from the fluorescence measurements. The blue line is the P2 molecule; The red line is the M2 molecule. Reproduced with permission from Ref. [50]. Copyright 2006 AAAS. **d** Structures of different chiral nanoparticles and the CD spectrum [53]. **e** Other structures of chiral nanoparticles [53]. Reproduced with permission from Ref. [53]. Copyright 2012 American Chemical Society

Toward the chirality detection, CD is one of the powerful methods. The absorption coefficients (*ε*) of the optically active substance to the left- and right-circular-polarized light composing the plane-polarized light are not equal, *ε*_L_ ≠ *ε*_R_, that is, it is circular dichroic. Barnes et al. [50] examined the circular polarization of the enantioisomers M2 and P2 (Fig. 3a). Representative fluorescence intensity traces of M2 and P2 excited by right- and left-handed circular-polarized laser radiation are plotted in Fig. 3b. The asymmetry factor *g* in the fluorescence detection CD signal is defined as 2[(*I*_L_ − *I*_R_)/(*I*_L_ + *I*_R_)], where *I*_R_ and *I*_L_ are fluorescence intensities under the excitation of right or left circular-polarized lights, respectively [51, 52]. The histograms in Fig. 3c show that the distributions of the asymmetric parameters *g* of individual M2 and P2 molecules appear as significant mirror images of each other. CD also sheds light on the chiral surface distortion of the nanomaterials. Govorov et al. [53] investigated the mechanism of the optical chirality of nanoparticles theoretically with the conclusion that the induced chirality was caused by harmonic mixing of the plasma due to the chiral surface distortion (Fig. 3d, e).

**Fig. 4:**
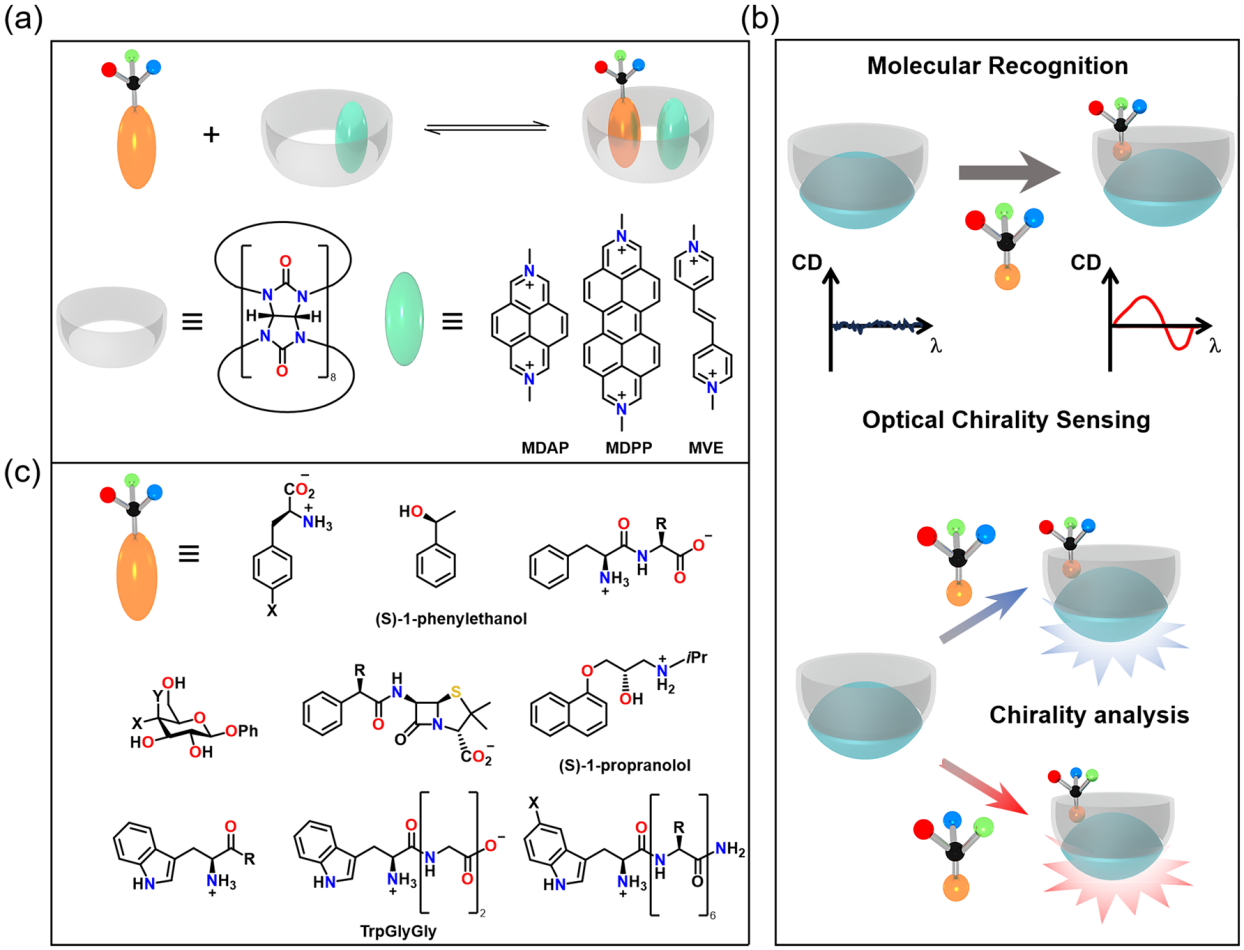
CD signal amplification with the assistance of other chromophores. **a** The chiroptical detection of binary complex [54]. **b** Illustration for the chiral supramolecular recognition. **c** The range of observable substrates [54]. Reproduced with permission from Ref. [54]. Copyright 2014 WILEY‐VCH

Considering that chiral molecules do not always possess strong chromophore and their CD signal can be rather weak in the far UV region, the chiral detection based on noncovalent interactions, metal coordination bonds and covalent bonds provide alternative options. The contact between the chiral group of the guest and the chromophore of the host can contribute to the molecular recognition, resulting in an induced CD signal. Biedermann and Nau [54] reported chiroptical detection of the binary complex in water (Fig. 4a) with a dicationic dye formed in cucurbit uril [8], which shows response in the CD spectra. Moreover, this chiral detector can be used to distinguish different analytes (Fig. 4b). The range of observable substrates is quite broad, including amino acids, peptides, proteins, pharmaceutical molecules, natural products, and small chiral organic molecules (Fig. 4c), which can go beyond the limits of functional groups excitingly. Note that this sensor requires that the chiral group should be similar to the neutral aromatic group, in case that efficient chiral transfer may not occur efficiently.

Polarized light can be further introduced to produce compounds of a particular chirality. Irradiation of circularly polarized light (CPL) is a promising approach to induct chirality and switch between different chiral isomers from achiral materials. As the chiral electromagnetic radiation, CPL can interact with chiral molecules selectively and enables enantioselective enrichment of prochiral photoexchange compounds [55–59]. In addition, to induce the chirality of self-organized materials, CPL has already been used in liquid crystals and polymers [60–63] as well as in solid [64], especially crystalline or nanoporous materials. Furthermore, it is possible for achiral molecules to construct chiral nanostructures by self-assembly when exerting different conditions including CPL irradiation [61, 65], and other external asymmetric stimulus [66–70].

**Fig. 5:**
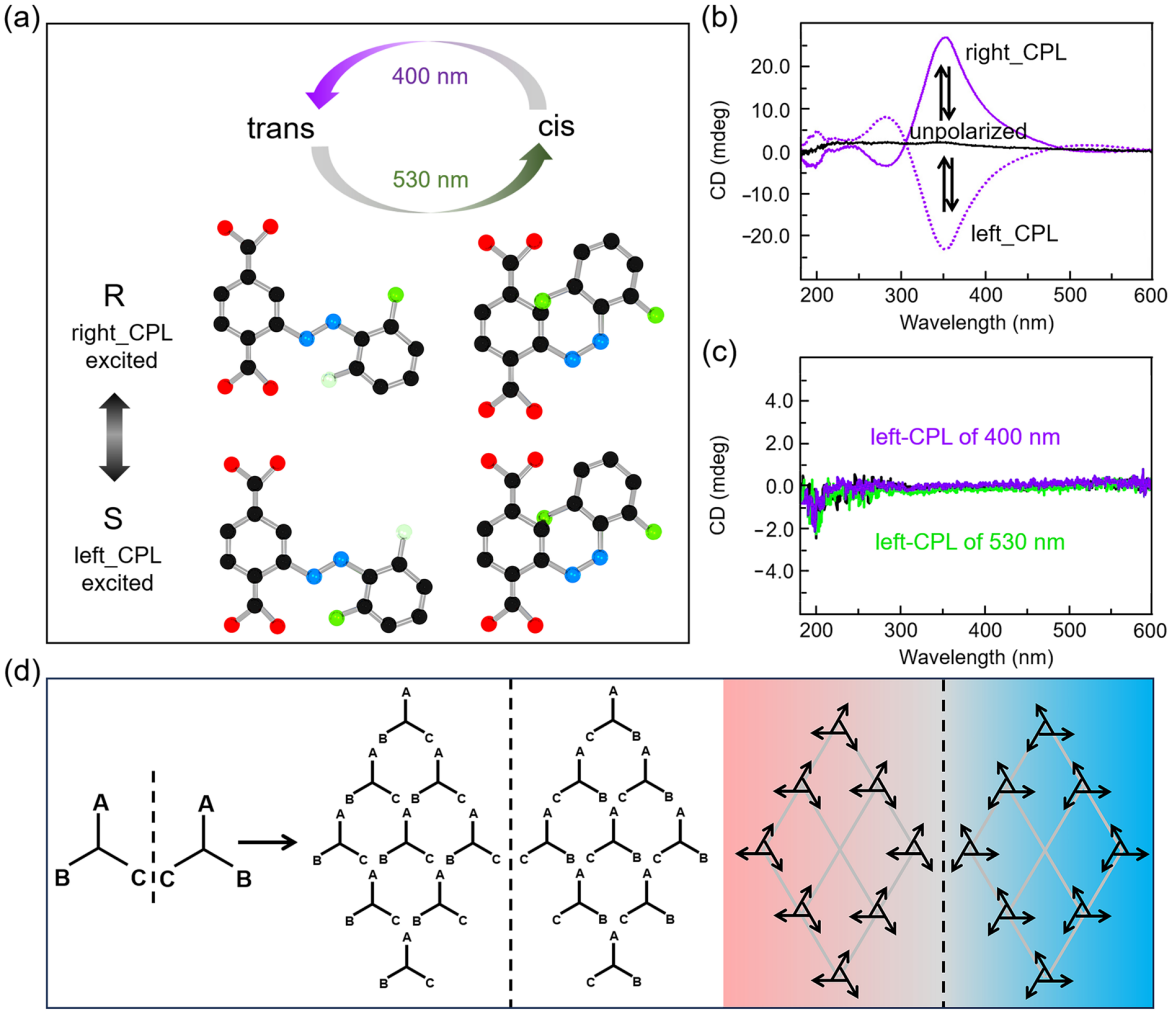
Asymmetric reaction induced by CPL. **a** Structures of photoswitchable fluorinated azobenzenes. The *trans*- to *cis*-conformation can be photoisomerized under green light and revert to *trans* under violet light, while R-*cis* and R-*trans* can be specially excited by right-CPL and vice versa [64]. **b** CD spectra of the fluorinated azobenzene attached MOF irradiated with unpolarized light (black lines), right-CPL (solid lines), and left-CPL (dotted lines) [64]. **c** CD spectra of the MOF without azobenzene side groups [64]. **d** Illustration for the assembly process of chiral compounds. Reproduced with permission from Ref. [64]. Copyright 2021 American Chemical Society

Heinke et al. [64] proposed a chiral solid with enantiomeric selective enrichment induced by CPL, where the structure is based on photoswitchable fluorinated azobenzene attached to the MOF. The *trans*- to *cis*-conformations can be photoisomerized under green light and revert to *trans* under violet light (Fig. 5a). There is no enantiomeric enrichment observed under unpolarized light, thus, the two isomers, R- and S-*trans* and R- and S-*cis*, were formed in equal amounts, respectively. However, chiral enrichment is caused under CPL. R-*trans* and R-*cis* enantiomers are activated under right CPL selectively, producing MOF of S-enantiomers enrichment, and vice versa (Fig. 5b, d). Different types of CPL favour different chiralities to produce compounds of a particular chirality. Especially, no enantiomeric enrichment is observed on the MOF films without azobenzene side groups under polarized light, demonstrating successful construction of symmetry-breaking transferring interfaces (Fig. 5c).

**Fig. 6:**
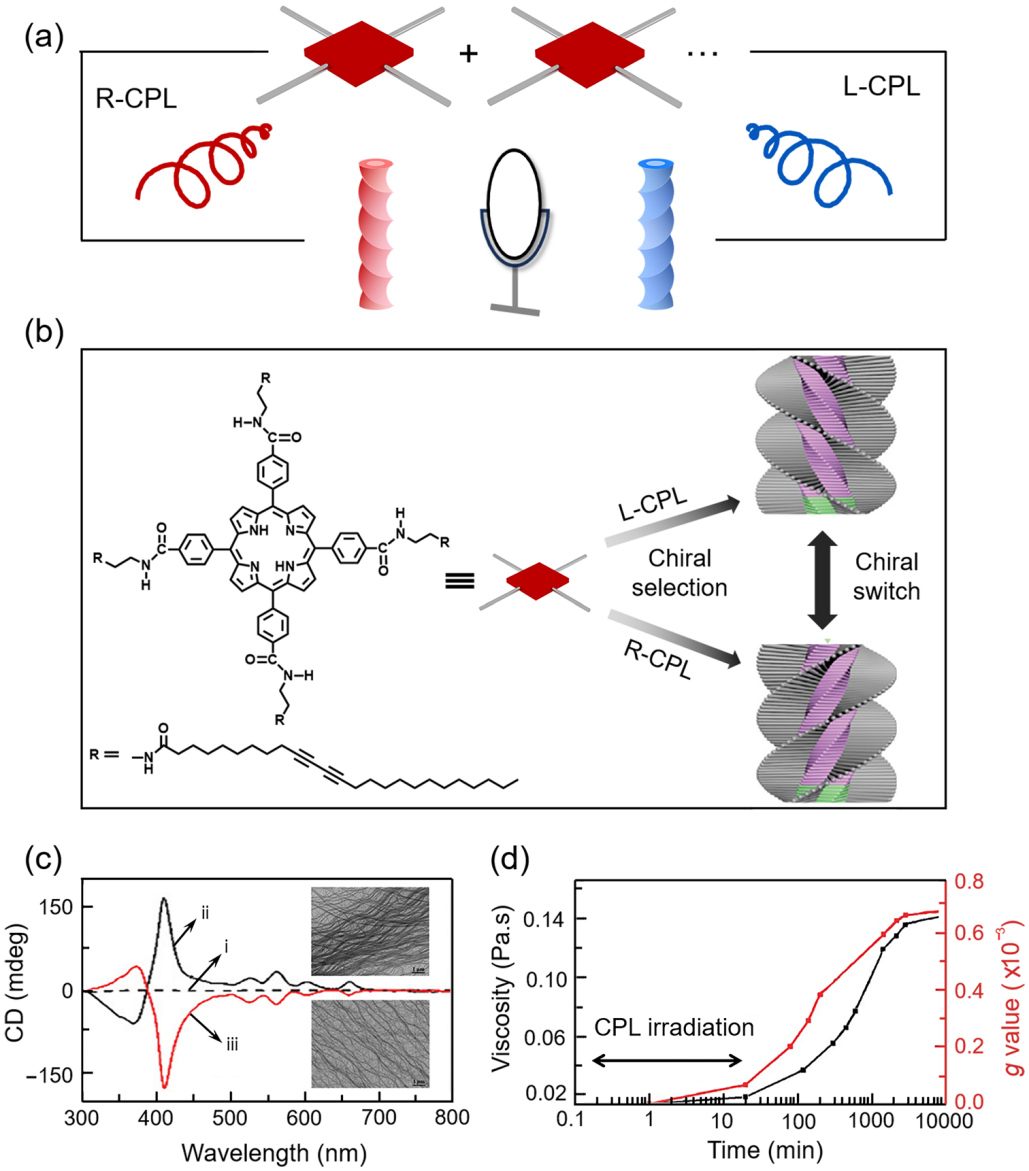
Asymmetric self-assembly induced by CPL. **a** CPL induced supramolecular assembly process** b** CPL induced protonation of the TPPDA and asymmetric stacking process [71]. **c** CD spectra and TEM images (inset) of different assemblies with two chiralities after irradiation with unpolarized UV (i), R-CPL (ii), and L-CPL (iii) for 20 min at the beginning [71]. **d** Time-resolved development of viscosity (black) and the *g*-factor (red) during the assembly process [71]. Reproduced with permission from Ref. [71]. Copyright 2019 RSC

In addition to inducing the molecular asymmetric reaction, the CPL was also adopted to construct the chiral supramolecular micro-structure. Zou et al. [71] proposed a kind of chiral self-assembly by achiral porphyrine molecules, TPPDA (Fig. 6a, b). It is necessary to notice that the CPL irradiation, which was applied at the beginning, provides the initial symmetric breaking of the whole process, although CPL irradiation had been abolished afterward. Furthermore, the chirality of the desired porphyrin composition can be stably locked by unpolarized ultraviolet irradiation. TEM characteristics showed that the porphyrin composition was bent after irradiation with R-CPL and L-CPL (Fig. 6c). No significant CD signal was detected for samples without UV treatment, or treated with unpolarized UV light. In detail, protonation of porphyrin promotes the symmetry breaking when the self-assembly is triggered by CPL, as electrostatic and *π*–*π* interactions of the cation–anion pair (protonated porphyrin moiety) may help to stabilize the further stack [65]. It is believed that the preferred enantiomeric form will be determined by the interaction of the angular momentum of CPL and π electrons in the porphyrin ring through the photoresolution effect [72], which can be contributed to the predominant different chiral stacking structure. The formed different chiral stacking structure will be acted as a chiral template and guide the assembly of the chiral supramolecular structure during the whole gelation process, giving rise to more chiral amplification and transfer after the removal of CPL irradiation. After the irradiation of CPL for 20 min, the *g*-value was measured as 6.7 × 10^−5^ in Fig. 6d, followingly, after the supramolecular self-assembly process occurred in the dark, the *g*-value increased to 7 × 10^−4^ with the enhancement of nearly tenfold. The high chiral stability was obtained because the supramolecular assembly chirality can be locked by polymerization of the surrounding diacetylene parts permanently.

The chiral detection and synthesis via CPL also have a few shortcomings. Firstly, this technique requires molecules with chromophore groups to describe the local characteristics. Secondly, the sample with purity of nearly 96% is required for polarized light detection, which makes it difficult to test mixed compounds, especially the in-situ chemical reaction. Thirdly, the specific regulation of CPL on the chirality remains unknown and it remains to be investigated for the perturbation of CPL on the chiral enrichment. The results of CPL on the reaction are usually discussed, while the microscopic mechanisms of the chiral response are rarely described in sufficient detail. Single-molecule platform may be a helpful method for the interaction between polarized light and chirality. A single molecule is connected between electrodes in single-molecule experiments, and the molecular characteristics can be described by detecting its electrical signal and other properties. This setup excludes ensemble-average effect and the influence of other impurity, which reflects the inherent interaction between the chiral molecule and polarized light, so as to further reveal the polarization-chirality relationship and provide certain guidance for the detection and induction of macroscopic polarization chirality.

## Molecular Chirality–Electronic Spin Interaction

**Fig. 7:**
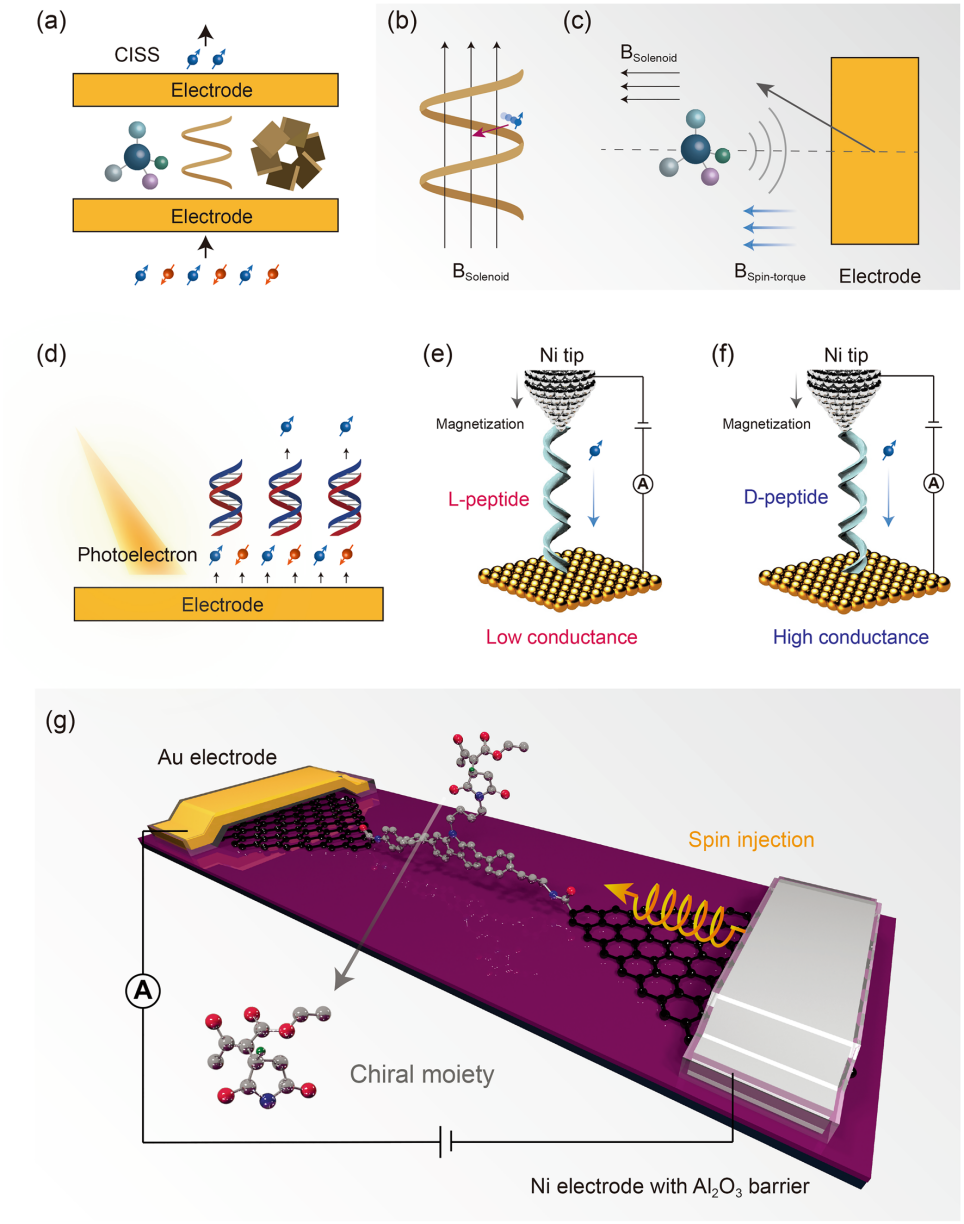
CISS effect and chirality detection by spin-polarized electron. **a** Schematic of the CISS effect [6]. **b** One theoretical model of the CISS effect. The solenoid magnetic field of the chiral molecule leads to the preference to a certain spin electron [7]. **c** Another theoretical model of the CISS effect. The solenoid magnetic field of the chiral molecule only provides the initial symmetry breaking, which induces the orbital magnetic moment of the electrode [76]. **d** Schematic of the spin-filtering of the photoelectron by the DNA on the metal surface.**e** The l-peptide leads to the filtering of the electrons which were spin-polarized by the downward magnetized Ni tip [77]. **f** The d-peptide leads to the transmitting of the electrons which were spin-polarized by the downward magnetized Ni tip [77]. **g** Schematic of the chirality detection during the reaction by the graphene-based single-molecule junction with spin injection [78]

As the other characteristics of the natural symmetry breaking, the spin of the electron also has a close relationship with molecular chirality, especially when the electron transmits through the molecule [7, 8, 73]. Although the electron is a part of the atom or molecule, its spin characteristics are usually not related with the molecular structure and the selectivity of chemical reaction (except the high spin induced acceleration by exchange correlation [74]). Therein, the main reason is that the spin angular momentum cannot be coupled with the free atom, molecule and chemical bond. The fixation to the molecule, e.g., crystallization, preparing the molecular layer or junction, provides an opportunity to couple the electron spin. The filter effect of the chiral molecular layer to the injected spin electron was firstly observed in 1999 [75]. In other words, the chiral molecules show a preference to the transmitted electrons with different spin (Fig. 7a, d) [6]. Similar chirality-induced spin selectivity (CISS) effect was also observed by the other electrode-molecule-electrode sandwich structure. Several theoretical models have been established for the CISS effect. An intuitive one is the pseudo magnetic field originated from the chiral molecule (especially a helix molecule, like DNA) to affect the moving electron with different spins. The linear momentum of the electron in the magnetic field was affected by its spin, and then lead to a spin-dependent transmission energy barriers (Fig. 7b) [7]. More specifically, one spin was favored, while the other was filtered. In fact, except from the helix molecule, a small chiral molecule could also show a strong CISS effect. Another theoretical model points out that the strong CISS effect can be mainly dominated by the metal electrodes. The small chiral molecule only provides a finite pseudo magnetic field as an initial symmetry breaking, which induces the orbital magnetic moment of metal electrode. With the spin–torque interaction between surface magnetization in the metal electrode and the spin imbalance in the chiral molecule, the same degree of CISS effect can also be observed (Fig. 7c) [76]. The physical origin of the CISS effect still exists many controversies, which also triggers the exploration of the origin of the symmetry breaking as well as the relationship.

Regardless, the known CISS effect would provide an opportunity to realize the precise and convenient chirality detection. The most sensitive technique, single-molecule detection, is also compatible with the spin injection. The one side of the electrode in the single-molecule junction can adopt a ferromagnetic (FM) metal to enable the injection of spin-polarized current via the magnetization. At the SPM setup, the peptide with different helical structure (L and D) can be trapped by the Au substrate and Ni tip (Fig. 7e, f). The degeneracy of its conductance was observed with determined magnetization direction of the Ni tip, and then enables the detection of the chirality without modification of the tip by a chiral partner [77]. More interestingly, the magnetization with opposite direction would cause the flip of the conductance, giving an opposite judgment criterion to the chirality. The sensitivity of this approach can be defined by calculating the polarization *P*:

$$P = \frac{{G_{{\text{H}}} - G_{{\text{L}}} }}{{G_{{\text{H}}} + G_{{\text{L}}} }}$$

where *G*_H_ and *G*_L_ are the high and low conductance levels for each enantiomers. The calculated polarization with *P*_L_ = 60% and *P*_D_ = 57% shows a high resolution for chirality detection. Here, we believe that this excellent ability should be pushed forward via the single-molecule technique and achieve more application. For example, the real-time monitoring of the chirality. The graphene-based junctions provide an opportunity owing to the high tolerance to the complex reaction environment and the compatibility to external stimulus. The spin-polarized electron was injected by external magnetized FM metal pattern and transmitted through the graphene electrode [78] (Fig. 7g). This setup enables the in situ monitoring of the variation of the chirality by CISS during the reaction, for example, the Michael addition between maleimide and ethyl acetoacetate. The reaction center was designed outside the main conduction path for further demonstrating the CISS mechanism. The “CISS polarization” (versus temperature and bias voltage), defined by $\frac{{J_{{{\text{up}}}} - J_{{{\text{down}}}} }}{{J_{{{\text{up}}}} + J_{{{\text{down}}}} }}$, was measured, where up/down describe the magnetization of the Ni electrode parallel/anti-parallel to the molecular axis. An unprecedented high CISS-polarization (up to > 50%) in a small-chiral-molecule based molecular junction was observed. According to the proposed theory [76], the outside chiral molecule could only provide an initial symmetry breaking by its pseudo magnetic field, which induces the orbital magnetic moment of Au electrode. With the interaction between surface magnetization in the Au electrode and the spin imbalance in the chiral molecule, the same degree of CISS effect can be observed. Therefore, the controversial mechanism of CISS effect was clarified, providing a new insight into the transferring between molecular chirality and electronic spin. This advance enables the in situ real-time monitoring to the chirality changes during the asymmetric reaction and the discoveries of the key chiral intermediates, the symmetry-breaking trajectories, the stereo-selective intermolecular interaction and the evolution of the asymmetric reaction trajectories at the single-molecule scale. We believe that the high integration of the single-molecule junction in future would pave the way to realize the real-time high-throughput detection of the asymmetric reactions based on the spintronics.

**Fig. 8:**
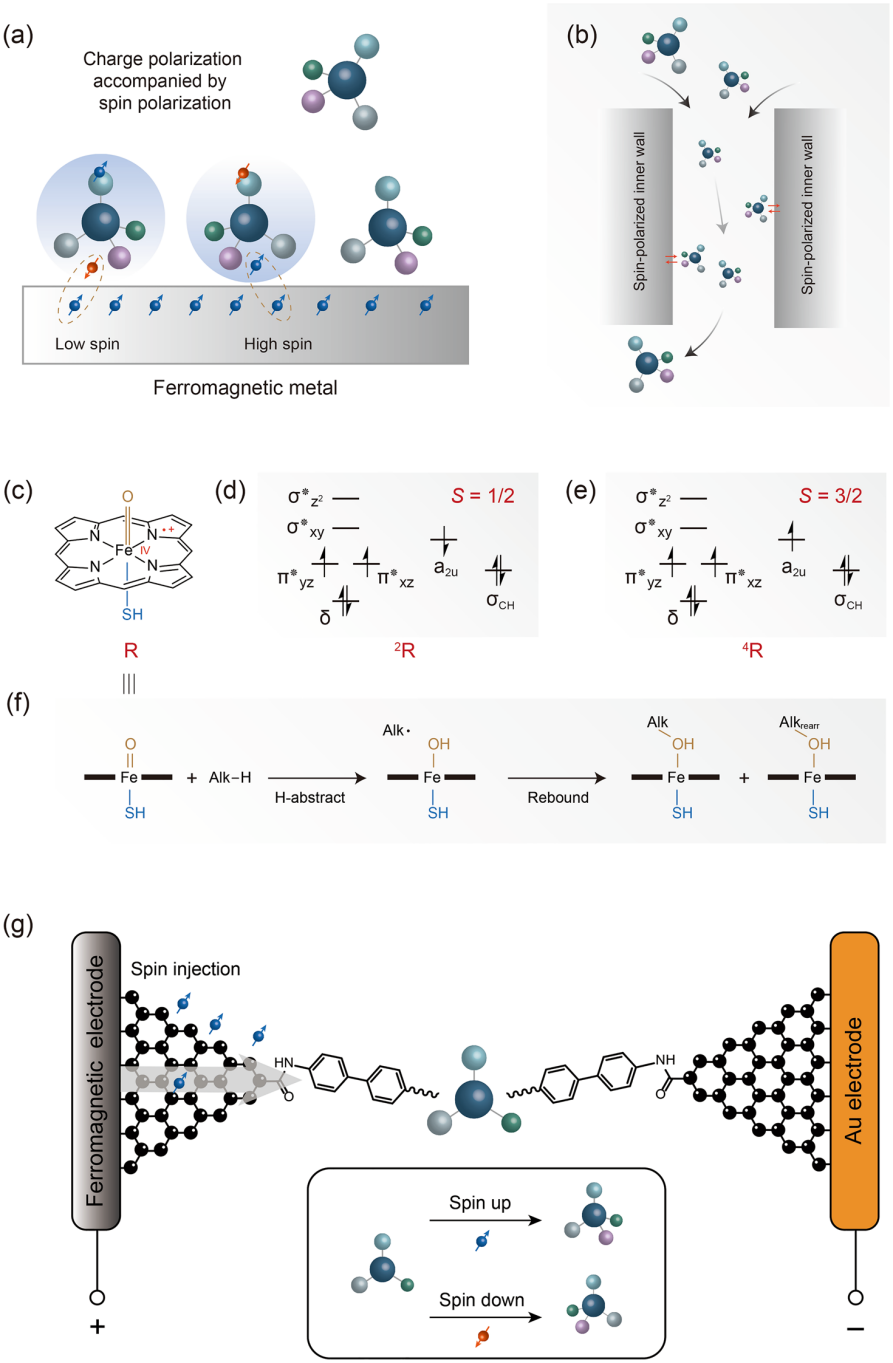
Chirality control by the spin polarization. **a** The magnetized metal surface leads to a selective absorption to the enantiomers [80]. **b** The strategy of adopting spin-polarized materials to resolve the chiral molecules. **c** Schematic of the porphyrin cation–radical oxo–iron [81]. **d** Diagram of the orbital occupancy of the low spin state of (**c**). **e** Diagram of the orbital occupancy of the high spin state of (**c**). **f** The reaction pathways of the oxygenation to alkene catalyzed by (**c**) [82]. **g** Schematic of the strategy to asymmetric catalysis by the spin polarization

In addition to the chirality detection, the chirality control by the spin electron is also fascinating. Along the line of the conventional strategies, we expect that the chirality control by spin characteristics can also be classified by the chirality resolution and direct asymmetric synthesis. For the former, the magnetized metal substrate affords the capability to absorb the chiral molecules selectively (Fig. 8a) [79], including DNA, peptide and amino acid, and then realize the resolution. The interaction between the molecules and substrate would cause a general electric dipole polarization. In this case, the chirality of the molecule renders this charge polarization accompanied by a spin polarization. With the magnetization of the FM metal substrate, the exchange interaction between the molecular spin and the spin of the substrate leads to a different absorption dynamics for enantiomers, and then realize the chirality resolution. This technique shows a high-performance resolution with an adsorption specificity up to more than 40% and it is expected to become a universal strategy. We believe that the column chromatography with electron spin-polarized fillers or inner wall is an important means to separate chiral molecules in the future (Fig. 8b).

The direct asymmetric synthesis “catalyzed” by spin electron may be more efficient and atomic economic. The electrocatalysis is a common technique for organic synthesis. However, spin characteristics of the electron was not widely used owing to the unfixed frame of the molecule and electron as discussed above. The spin-dependent reaction dynamics should be firstly highlighted here, as the basis of further exploration. The different spin states of the reaction center usually render different pathways and corresponding selectivity. The cytochrome P450 enzymes contain a porphyrin cation–radical oxo–iron center (Por^**·**+^Fe^IV^ = O) (Fig. 8c) [80], which has three unpaired electrons. Considering the spin of these three electrons, both the initial doublet- (*S* = 1/2, Fig. 8d) and quartet- (*S* = 3/2, Fig. 8e) states can be obtained, and contribute to different oxygenation products of alkene. More specifically, after the activation to C–H bond, the subsequent concerted low-spin pathway leads to a hydroxylation product via the rebound of the alkyl, while the stepwise high-spin pathway leads to a rearranged product (Fig. 8f). Another example related with the electrocatalysis is the electrochemical splitting of water to prepare hydrogen and oxygen. The combination of OH^·^ intermediate to form H_2_O_2_ causes a higher overpotential. The issue can be addressed by modifying a chiral layer at anode, which led to a homo spin of the electron on OH· and then suppressed their combination [81]. Thus, the spin characteristics could be regarded as a powerful tool to regulate the chemical reaction, which should also include the chirality. The spin states of the integrated molecule in junction can be manipulated efficiently by the mechanical force and gate/bias voltages, thus giving a significant insight to the chirality control. In addition, we also expected that the strict fixation of the focused prochiral molecule in junctions may couple the spin characteristics of electron. More specifically, the rigid molecular bridge and stable anchor interface with electrodes should be adopted (Fig. 8g). The setup enables the efficient interaction between the determined spatial direction and spin-polarized electrode. The different thermodynamic or kinetic preference to the pathway involving different chiralities may be achieved. The replaceability of the functional center on the molecular bridge provides a universal strategy. The high integration of the junction raises the possibility of a large-scale asymmetric preparation.

With the exception of the spin polarization, the single-molecule junction also enables the manipulation of the fixed molecule by electric field. More specifically, for example, the distortion of the molecule, i.e., the control of the conformation, can be realized by an applied bias voltage[82, 83]. It is well-known that the conformation of the prochiral center is the key factor to determine the subsequent asymmetric pathways. In combination with the precise detection and control of the molecular conformation, the well-designed single-molecule device also provides a basement for the asymmetric synthesis.

## Origin of Chirality

The relationship among the symmetry breaking inspires us to detect and control the chirality by spin electron and polarized light, which also prompts the deliberation of the origin of these symmetry breakings, especially the homochirality on earth. Mimicking the nature has the potential to achieve the chirality detection and control fundamentally. The CPL from extraterrestrials and the excessive l-amino acids in meteorites are likely to create the homochirality of life on the early earth [1]. Moreover, other theoretical models have also been established. A typical example is the sergeants-and-soldiers effect. The auto-catalysis by the chiral molecule would cause the symmetry breaking of the whole system. Therefore, a deeper understanding from the single-molecule perspective is necessary. When a single-molecule “sergeant” being focused in junction, the chiral amplification may be characterized directly, which further guide the macroscopic asymmetric synthesis. Another theoretical model demonstrates that the symmetry is an equilibrium state but not stable [84]. Therefore, the external energy could drive the state far from equilibrium and render the random symmetry breaking and chiral enrichment. The detailed chemical picture is the cross-correlation among the interlocked individual reaction patterns. This highlights the consideration of not only one-molecule reactions but the collective effect of multiple molecules. The origin of the chirality is still a basic but unaddressed issue. We believe that any progress in the exploration will provide the guidance to detect and control the chirality more effectively.

## Summary

Trapping of a single molecule, especially covalently integrating it into the nanogap of electrodes, affords an effective interactive interface among molecular chirality, electronic spin and photonic polarization. This enables the universal chiral recognition, control and synthesis via the input of the asymmetric variables (e.g., electronic spin and photonic polarization). Through close collaborations between chemists, physicists, materials scientists, and engineers, the integration from a single-molecule device to high-density device arrays may lay the foundation for precise high-throughput synthesis with single-molecule resolution. In addition to this, focusing on one individual molecule breaks the ensemble-average effect, so as to further reveal the detailed physic picture of asymmetric transformation and the origin of the chirality, which will invite intense researches in the future.

## References

[1] Bailey J, Chrysostomou A, Hough JH, Gledhill TM, Mccall A (1998). Circular polarization in star-formation regions: implications for biomolecular homochirality. Science.

[2] Fub W, Chrysostomou A, Hough JH, Gledhill TM, Mccall A (2009). Does life originate from a single molecule?. Chirality.

[3] Hsieh D, Xia Y, Wray L, Qian D, Pal A (2009). Observation of unconventional quantum spin textures in topological insulators. Science.

[4] Kim YH, Zhai Y, Lu H, Pan X, Xiao C (2021). Chiral-induced spin selectivity enables a room-temperature spin light-emitting diode. Science.

[5] Sharma V, Crne M, Park JO, Srinivasarao M (2009). Structural origin of circularly polarized iridescence in jeweled beetles. Science.

[6] Gohler B, Hamelbeck V, Markus TZ, Kettner M, Hanne GF (2011). Spin selectivity in electron transmission through self-assembled monolayers of double-stranded DNA. Science.

[7] Naaman R, Paltiel Y, Waldeck DH (2019). Chiral molecules and the electron spin. Nat. Rev. Chem..

[8] Brandt JR, Salerno F, Fuchter MJ (2017). The added value of small-molecule chirality in technological applications. Nat. Rev. Chem..

[9] Fiederling R, Keim M, Reuscher G, Ossau W, Schmidt G (1999). Injection and detection of a spin-polarized current in a light-emitting diode. Nature.

[10] Kikkawa JM, Awschalom DD (2000). All-optical magnetic resonance in semiconductors. Science.

[11] Li Y, Yang C, Guo X (2020). Single-molecule electrical detection: a promising route toward the fundamental limits of chemistry and life science. Acc. Chem. Res..

[12] Barkai E, Jung YJ, Silbey R (2004). Theory of single-molecule spectroscopy: beyond the ensemble average. Annu. Rev. Phys. Chem..

[13] Lu HP, Xun LY, Xie XS (1998). Single-molecule enzymatic dynamics. Science.

[14] Rust MJ, Bates M, Zhuang X (2006). Sub-diffraction-limit imaging by stochastic optical reconstruction microscopy (STORM). Nat. Methods.

[15] Zrimsek AB, Chiang N, Mattei M, Zaleski S, McAnally MO (2017). Single-molecule chemistry with surface- and tip-enhanced raman spectroscopy. Chem. Rev..

[16] Heller I, Hoekstra TP, King GA, Peterman EJG, Wuite GJL (2014). Optical tweezers analysis of DNA–protein complexes. Chem. Rev..

[17] Armani AM, Kulkarni RP, Fraser SE, Flagan RC, Vahala KJ (2007). Label-free, single-molecule detection with optical microcavities. Science.

[18] Deamer D, Akeson M, Branton D (2016). Three decades of nanopore sequencing. Nat. Biotechnol..

[19] Xin N, Guan J, Zhou C, Chen X, Gu C (2019). Concepts in the design and engineering of single-molecule electronic devices. Nat. Rev. Phys..

[20] Xiang D, Wang X, Jia C, Lee T, Guo X (2016). Molecular-scale electronics: from concept to function. Chem. Rev..

[21] Verbrugge S, Lansky Z, Peterman EJG (2009). Kinesin's step dissected with single-motor FRET. Proc. Natl. Acad. Sci. U.S.A..

[22] Florin EL, Moy VT, Gaub HE (1994). Adhesion forces between individual ligand-receptor pairs. Science.

[23] Liu CM, Kubo K, Wang E, Han K, Yang F (2017). Single polymer growth dynamics. Science.

[24] Xu B, Tao N (2003). Measurement of single-molecule resistance by repeated formation of molecular junctions. Science.

[25] Reed MA, Zhou C, Muller CJ, Burgin TP, Tour JM (1997). Conductance of a molecular junction. Science.

[26] Liang W, Shores MP, Bockrath M, Long JR, Park H (2002). Kondo resonance in a single-molecule transistor. Nature.

[27] Kubatkin S, Danilov A, Hjort M, Cornil J, Brédas J (2003). Single-electron transistor of a single organic molecule with access to several redox states. Nature.

[28] Jia C, Ma B, Xin N, Guo X (2015). Carbon electrode–molecule junctions: a reliable platform for molecular electronics. Acc. Chem. Res..

[29] Park J, Pasupathy AN, Goldsmith JI, Chang C, Yaish Y (2002). Coulomb blockade and the Kondo effect in single-atom transistors. Nature.

[30] Cao Y, Dong S, Liu S, He L, Gan L (2012). Building high-throughput molecular junctions using indented graphene point contacts. Angew. Chem. Int. Ed..

[31] Wu Y, Xiao Y, Wang X, Li X, Wang Y (2019). Chirality discrimination at the single molecule level by using a cationic supermolecule quasi-gated organic field effect transistor. ACS Sens..

[32] Salikolimi K, Praveen VK, Sudhakar AA, Yamada K, Nishizawa Horimoto N (2020). Helical supramolecular polymers with rationally designed binding sites for chiral guest recognition. Nat. Commun..

[33] Das S, Xu S, Ben T, Qiu S (2018). Chiral recognition and separation by chirality-enriched metal–organic frameworks. Angew. Chem. Int. Ed..

[34] Li B, Yang X, Wu X, Luo Z, Zhong C (2007). Enantioselective recognition for carboxylic acids by novel chiral macrocyclic polyamides derived from l-/d-tartaric acid. Supramol. Chem..

[35] Okamoto Y, Yashima E (1998). Polysaccharide derivatives for chromatographic separation of enantiomers. Angew. Chem. Int. Ed..

[36] Labuta J, Ishihara S, Šikorský T, Futera Z, Shundo A (2013). NMR spectroscopic detection of chirality and enantiopurity in referenced systems without formation of diastereomers. Nat. Commun..

[37] Zhang L, Wang G, Xiong C, Zheng L, He J (2018). Chirality detection of amino acid enantiomers by organic electrochemical transistor. Biosens. Bioelectron..

[38] Rekharsky M, Inoue Y (2000). Chiral recognition thermodynamics of beta-cyclodextrin: the thermodynamic origin of enantioselectivity and the enthalpy–entropy compensation effect. J. Am. Chem. Soc..

[39] Okamoto Y, Ikai T (2008). Chiral HPLC for efficient resolution of enantiomers. Chem. Soc. Rev..

[40] Kreituss I, Bode JW (2017). Flow chemistry and polymer-supported pseudoenantiomeric acylating agents enable parallel kinetic resolution of chiral saturated N-heterocycles. Nat. Chem..

[41] Steffensen MB, Rotem D, Bayley H (2014). Single-molecule analysis of chirality in a multicomponent reaction network. Nat. Chem..

[42] Jia W, Hu C, Wang Y, Liu Y, Wang L (2022). Identification of single-molecule catecholamine enantiomers using a programmable nanopore. ACS Nano.

[43] Aviram A, Ratner MA (1974). Molecular rectifiers. Chem. Phys. Lett..

[44] Xin N, Wang J, Jia C, Liu Z, Zhang X (2017). Stereoelectronic effect-induced conductance switching in aromatic chain single-molecule junctions. Nano Lett..

[45] Su TA, Neupane M, Steigerwald ML, Venkataraman L, Nuckolls C (2016). Chemical principles of single-molecule electronics. Nat. Rev. Mater..

[46] Liu Z, Li X, Masai H, Huang X, Tsuda S (2021). A single-molecule electrical approach for amino acid detection and chirality recognition. Sci. Adv..

[47] Nishino T, Umezawa Y (2008). Single-molecule chiral recognition on a surface by chiral molecular tips. Anal. Chem..

[48] Arago DFJ, Fresnel AJ (1819). On the action of rays of polarized light upon each other. Ann. Chim. Phys..

[49] Silliman RH (1974). Fresnel and the emergence of physics as a discipline. Hist. Stud. Phys. Sci..

[50] Hassey R, Swain EJ, Hammer NI, Venkataraman D, Barnes MD (2006). Probing the chiroptical response of a single molecule. Science.

[51] Richardson FS, Riehl JP (1977). Circularly polarized luminescence spectroscopy. Chem. Rev..

[52] Riehl JP, Richardson FS (1986). Circularly polarized luminescence spectroscopy. Chem. Rev..

[53] Fan Z, Govorov AO (2012). Chiral nanocrystals: plasmonic spectra and circular dichroism. Nano Lett..

[54] Biedermann F, Nau WM (2014). Noncovalent chirality sensing ensembles for the detection and reaction monitoring of amino acids, peptides, proteins, and aromatic drugs. Angew. Chem. Int. Ed..

[55] Rijeesh K, Hashim PK, Noro S, Tamaoki N (2015). Dynamic induction of enantiomeric excess from a prochiral azobenzene dimer under circularly polarized light. Chem. Sci..

[56] Hashim PK, Tamaoki N (2019). Enantioselective photochromism under circularly polarized light. ChemPhotoChem.

[57] Bisoyi HK, Li Q (2014). Light-directing chiral liquid crystal nanostructures: from 1D to 3D. Acc. Chem. Res..

[58] Hashim PK, Thomas R, Tamaoki N (2011). Induction of molecular chirality by circularly polarized light in cyclic azobenzene with a photoswitchable benzene rotor. Chem. Eur. J..

[59] Huck NPM, Jager WF, de Lange B, Feringa BL (1996). Dynamic control and amplification of molecular chirality by circular polarized light. Science.

[60] Burnham KS, Schuster GB (1999). Transfer of chirality from circularly polarized light to a bulk material property: propagation of photoresolution by a liquid crystal transition. J. Am. Chem. Soc..

[61] Iftime G, Labarthet FL, Natansohn A, Rochon P (2000). Control of chirality of an azobenzene liquid crystalline polymer with circularly polarized light. J. Am. Chem. Soc..

[62] Li J, Schuster GB, Cheon K-S, Green MM, Selinger JV (2000). Switching a helical polymer between mirror images using circularly polarized light. J. Am. Chem. Soc..

[63] Hore DK, Natansohn AL, Rochon PL (2003). The characterization of photoinduced chirality in a liquid-crystalline azo polymer on irradiation with circularly polarized light. J. Phys. Chem. B.

[64] Kanj AB, Bürck J, Vankova N, Li C, Mutruc D (2021). Chirality remote control in nanoporous materials by circularly polarized light. J. Am. Chem. Soc..

[65] Kim J, Lee J, Kim WY, Kim H, Lee S (2015). Induction and control of supramolecular chirality by light in self-assembled helical nanostructures. Nat. Commun..

[66] Ribó JM, Crusats J, Sagués F, Claret J, Rubires R (2001). Chiral sign induction by vortices during the formation of mesophases in stirred solutions. Science.

[67] Sun J, Li Y, Yan F, Liu C, Sang Y (2018). Control over the emerging chirality in supramolecular gels and solutions by chiral microvortices in milliseconds. Nat. Commun..

[68] Rikken GLJA, Raupach E (2000). Enantioselective magnetochiral photochemistry. Nature.

[69] Micali N, Engelkamp H, van Rhee PG, Christianen PCM, Monsù Scolaro L (2012). Selection of supramolecular chirality by application of rotational and magnetic forces. Nat. Chem..

[70] Xu Y, Yang G, Xia H, Zou G, Zhang Q (2014). Enantioselective synthesis of helical polydiacetylene by application of linearly polarized light and magnetic field. Nat. Commun..

[71] Hu J, Xie Y, Zhang H, He C, Zhang Q (2019). Chiral induction, modulation and locking in porphyrin based supramolecular assemblies with circularly polarized light. Chem. Commun..

[72] Tang Y, Cohen AE (2010). Optical chirality and its interaction with matter. Phys. Rev. Lett..

[73] Gersten J, Kaasbjerg K, Nitzan A (2013). Induced spin filtering in electron transmission through chiral molecular layers adsorbed on metals with strong spin-orbit coupling. J. Chem. Phys..

[74] Shaik S, Chen H, Janardanan D (2010). Exchange-enhanced reactivity in bond activation by metal–oxo enzymes and synthetic reagents. Nat. Chem..

[75] Ray K, Ananthavel SP, Waldeck DH, Naaman R (1999). Asymmetric scattering of polarized electrons by organized organic films of chiral molecules. Science.

[76] Alwan S, Dubi Y (2021). Spinterface origin for the chirality-induced spin-selectivity effect. J. Am. Chem. Soc..

[77] Aragones AC, Medina E, Ferrer-Huerta M, Gimeno N, Teixidó M (2017). Measuring the spin-polarization power of a single chiral molecule. Small.

[78] Yang C, Li Y, Zhou S, Guo Y, Jia C (2023). Real-time monitoring of reaction stereochemistry through single-molecule observations of chirality-induced spin selectivity. Nat. Chem..

[79] Banerjee-Ghosh K, Dor OB, Tassinari F, Capua E, Yochelis S (2018). Separation of enantiomers by their enantiospecific interaction with achiral magnetic substrates. Science.

[80] Shaik S, Hirao H, Kumar D (2007). Reactivity of high-valent iron-oxo species in enzymes and synthetic reagents: a tale of many states. Acc. Chem. Res..

[81] Mtangi W, Tassinari F, Vankayala K, Jentzsch AV, Adelizzi B (2017). Control of electrons' spin eliminates hydrogen peroxide formation during water splitting. J. Am. Chem. Soc..

[82] Harzmann GD, Frisenda R, van der Zant HSJ, Mayor M (2015). Single-molecule spin switch based on voltage-triggered distortion of the coordination sphere. Angew. Chem. Int. Ed..

[83] Meng L, Xin N, Wang J, Xu J, Ren S (2021). Atomically precise engineering of single-molecule stereoelectronic effect. Angew. Chem. Int. Ed..

[84] Piñeros WD, Tlusty T (2022). Spontaneous chiral symmetry breaking in a random driven chemical system. Nat. Commun..

